# CRISPR and the Promise of Gene Editing: Unlocking Curative Possibilities for Liver Disorders Worldwide

**DOI:** 10.14309/crj.0000000000002061

**Published:** 2026-03-30

**Authors:** Sabah Kulsum, Sajjad Ahmed Khan, Hema Sameera Pinnam, Harshita Malik, Hassam Ali, Dushyant Singh Dahiya

**Affiliations:** 1 Department of Internal Medicine, New York Medical College-StMary's StClare's, New York, NY; 2 Department of Internal Medicine, Birat Medical College Teaching Hospital, Tankisinuwari, Nepal; 3 Department of Internal Medicine, JSS Medical College, Mysore, Karnataka, India; 4 Department of Internal Medicine, Kasturba Medical College, Manipal, Karnataka, India; 5 Department of Gastroenterology, Hepatology and Nutrition, East Carolina University/Brody School of Medicine, Greenville, NC; 6 Division of Gastroenterology, Hepatology & Motility, The University of Kansas School of Medicine, Kansas City, KS

**Keywords:** gastroenterology, hepatology, gene therapy, liver

## INTRODUCTION: A TURNING POINT IN HEPATOLOGY

Liver diseases continue to impose a significant global health burden, accounting for millions of deaths every year due to viral hepatitis, cirrhosis, hepatocellular carcinoma (HCC), and a wide range of inherited metabolic disorders.^1^ Although remarkable progress has been made in antiviral therapy, endoscopic interventions, and transplantation medicine, the reality remains that many liver conditions are chronic, progressive, and only partially treatable.^2^ The dream of curative therapy, once an aspiration reserved for science fiction, is now entering the realm of possibility with the advent of Clustered Regularly Interspaced Short Palindromic Repeats (CRISPR)-based gene editing. This revolutionary technology holds the potential not just to manage liver disease but to fundamentally rewrite the biological codes that drive them.

## WHY THE LIVER IS IDEAL FOR GENE EDITING

The liver possesses unique characteristics that make it one of the most accessible and responsive organs for genetic manipulation. Its rich vascularity facilitates the efficient delivery of gene-editing tools via systemic administration.^3^ Moreover, hepatocytes exhibit robust regenerative capacity, enabling them to incorporate edited genetic material and proliferate—amplifying the therapeutic impact.^3^

The liver also plays a central role in metabolism and detoxification, meaning that genetic defects in hepatic pathways can result in severe systemic disease.^4^ The ability to modify these pathways directly at the genomic level provides a powerful opportunity to cure conditions that were previously managed only through lifelong medication or transplantation. As a result, the liver has become a major focus of early-phase clinical trials examining the promise of CRISPR-Cas systems for therapeutic use.

## BREAKTHROUGHS IN CLINICAL GENE EDITING: PROOF OF CONCEPT

One of the most significant milestones in human gene editing was achieved when a CRISPR-based therapy was administered intravenously to patients with transthyretin (TTR) amyloidosis.^5^ A single dose resulted in a dramatic and sustained reduction in circulating TTR levels—offering the first real proof that in vivo gene editing can produce meaningful clinical outcomes in humans. This breakthrough demonstrated the durability and precision of hepatic gene editing and ignited global interest in extending CRISPR to a wider range of liver diseases.

Current research focuses on 3 major domains: monogenic metabolic disorders, viral hepatitis, and liver malignancies. Each presents distinct opportunities and challenges, yet all stand to benefit from the power of CRISPR to modify or silence pathogenic genes directly.

## MONOGENIC LIVER DISORDERS: A PATHWAY TOWARD CURATIVE THERAPY

Inherited liver diseases, many caused by single-gene mutations, represent some of the most promising candidates for CRISPR therapy. Conditions like Wilson disease, familial hypercholesterolemia, hemophilia, and alpha-1 antitrypsin deficiency often lead to irreversible complications if untreated. Traditional therapies aim only to control symptoms; gene editing offers a pathway to an actual cure by correcting or disabling defective genetic sequences.

Early animal studies have shown encouraging results. For example, CRISPR-mediated correction of alpha-1 antitrypsin mutations in mouse models restored normal protein function and prevented hepatocyte injury.^6^ Similar success has been seen in models of metabolic storage diseases. As technology matures, clinical trials for these disorders are expected to expand rapidly.

## CRISPR AGAINST VIRAL HEPATITIS: THE CHALLENGE OF cccDNA

Chronic hepatitis B virus (HBV) infection remains one of the world's most pressing liver health issues, affecting nearly 257 million people globally.^7^ Despite advances in antiviral therapy, achieving a true cure has remained elusive due to the persistence of covalently closed circular DNA (cccDNA) in hepatocyte nuclei.^8^ This viral reservoir ensures ongoing replication even in patients who achieve viral suppression.

CRISPR has introduced the possibility of directly targeting and destroying HBV cccDNA. Laboratory experiments have shown that guide RNAs can be designed to precisely cut viral DNA sequences, thereby disrupting replication.^9^ If this strategy proves safe and effective in humans, it could represent a monumental leap toward the long-sought functional cure of HBV. Emerging research is also exploring CRISPR applications in hepatitis D and E, as well as in host-factor modification to enhance antiviral immunity.^10^

## CRISPR IN HEPATOCELLULAR CARCINOMA: REINVENTING CANCER THERAPY

Hepatocellular carcinoma is a rapidly growing global health threat.^11^ Although immunotherapies and targeted agents have improved outcomes, survival remains limited for many patients. CRISPR presents a novel frontier in oncology by enabling precise editing of tumor-associated genes and enhancing the tumor-killing potential of immune cells.^12^

Researchers are developing CRISPR-engineered CAR-T cells and CRISPR-modified natural killer cells capable of infiltrating liver tumors and overcoming immune evasion mechanisms.^13^ In addition, CRISPR-based strategies can be used to knock out genes that drive tumor growth, metastasis, or resistance to therapy. Although most of these approaches remain in preclinical stages, they represent a bold reimagining of liver cancer treatment.^13^

## GENE THERAPY TARGETS IN PEDIATRIC LIVER DISEASES

It is also important to acknowledge that many established and emerging targets for gene therapy are pediatric-onset disorders, particularly in the context of inborn errors of metabolism and other monogenic diseases.^14^ Several of these conditions, including urea cycle disorders, glycogen storage diseases, lysosomal storage disorders, and inherited cholestatic liver diseases, manifest early in life and account for a substantial proportion of candidates for liver-directed gene therapy.^15^ These disorders are comparatively underrepresented in adult-focused discussions, despite strong biological rationale and encouraging preclinical and early clinical data supporting gene-based interventions. Expanding the scope to include pediatric-onset metabolic and genetic liver diseases provides a more comprehensive and balanced perspective on the current and future applications of gene therapy across the lifespan.

## ETHICAL, SAFETY, AND EQUITY CHALLENGES

Despite its transformative potential, CRISPR technology brings profound ethical and safety concerns. Off-target editing, where unintended genetic sites are modified, poses a risk of unpredictable consequences, including carcinogenesis. Long-term follow-up data are limited, and the durability of gene edits across decades remains uncertain. Moreover, immune reactions to viral vectors used for CRISPR delivery require careful study.^16^

Ethically, gene editing must be restricted to somatic cells to avoid germline transmission, which carries far-reaching moral and societal implications. Regulatory frameworks must evolve to ensure responsible use, safety monitoring, transparency, and equitable distribution of gene-editing therapies.^17^

Access is a critical issue. The populations most affected by liver diseases—particularly HBV, HCC, and metabolic disorders—often reside in low- and middle-income countries. If CRISPR technologies are to truly transform global health, they must be developed and delivered in ways that do not exacerbate existing health care disparities.^18^

## CURRENT GENE-EDITING APPROACHES BEYOND CRISPR

Beyond CRISPR–Cas systems, other gene-editing technologies such as zinc finger nucleases (ZFNs), transcription activator-like effector nucleases (TALENs), and emerging base and prime editing approaches are under investigation. Although ZFNs and TALENs enable targeted genome modification, their clinical application is limited by complex protein engineering, reduced scalability, and higher costs. Base and prime editing allow precise nucleotide changes without inducing double-strand DNA breaks but remain technically challenging and are at relatively early stages of in vivo development.^19^ In contrast, CRISPR offers ease of design, high targeting flexibility, multiplexing capability, and rapidly advancing delivery systems, positioning it as the most promising and clinically translatable gene-editing platform for liver-directed therapies at present.

## THE ROAD AHEAD: FROM MANAGEMENT TO MOLECULAR CURE

The rapid pace of CRISPR innovation signals a paradigm shift in hepatology. We are moving from an era centered on disease control and symptom management to one aimed at precision correction and cure. Although many obstacles remain, the accelerating progress in delivery systems, editing accuracy, and safety profiling suggests that CRISPR will soon transition from an experimental intervention to a mainstream therapeutic option. Interdisciplinary collaboration among hepatologists, geneticists, molecular biologists, bioethicists, and policymakers will be essential to guide this transition responsibly. With thoughtful development and global cooperation, CRISPR has the potential to redefine the future of liver disease care.

## CONCLUSION

CRISPR and gene editing represent one of the most profound scientific revolutions of our generation. For liver disease—long dominated by chronic management and limited curative options—the emergence of precise, durable genomic modification is ushering in a new era of possibility. Although challenges persist, the trajectory is unmistakably forward. Each new breakthrough moves us closer to a world where liver diseases can not only be treated but truly cured.

## DISCLOSURES

Author contributions: Conceptualization: S.K., S.A.K., and D.S.D. Literature search: H.S.P. and H.M. Manuscript writing-original draft: S.K., S.A.K., H.S.P., H.M., H.A., and D.S.D. Manuscript writing-review and editing: H.A. and D.S.D.

Financial disclosure: None to report.

Conflicts of Interests: None to report.

## ABBREVIATIONS:

cccDNA: Covalently closed circular DNA
CRISPR: Clustered Regularly Interspaced Short Palindromic Repeats
DNA: Deoxyribonucleic acid
HBV: Hepatitis B virus
HCC: Hepatocellular carcinoma
RNA: Ribonucleic acid
TTR: Transthyretin
CAR-T: Chimeric Antigen Receptor-T
ZFNs: Zinc finger nucleases
TALENs: Transcription activator-like effector nucleases
